# MRI Findings of Desmoplastic Infantile Ganglioglioma: A Case Report and Analysis

**DOI:** 10.7759/cureus.43770

**Published:** 2023-08-19

**Authors:** Diego Jordão L Dias, Amanda Selvátici dos S Dias, Marcos V Camargo, Henrique M Lederman

**Affiliations:** 1 Neuroradiology, Hospital de Base - Faculdade de Medicina de São José do Rio Preto (FAMERP), São José do Rio Preto, BRA; 2 Neurology, Hospital de Base - Faculdade de Medicina de São José do Rio Preto (FAMERP), São José do Rio Preto, BRA; 3 Pediatric Radiology, Institute of Pediatric Oncology – GRAACC – Universidade Federal de São Paulo (UNIFESP), São Paulo, BRA

**Keywords:** benign tumors, bulging fontanelle, childhood central nervous system tumor, intracranial tumor, mri brain and spine, neuro radiology, desmoplastic infantile tumor, brain oncology

## Abstract

Desmoplastic infantile gangliogliomas (DIG) are rare intracranial tumors that predominantly affect children. They are characterized by a mixture of glial and neuronal components interspersed with abundant fibrous stroma and are typically located on the surface of the cerebral hemispheres. In this case report, we present a seven-year-old male child with a late presentation of DIG, which is typically diagnosed between zero and 60 months of age. We discuss the MRI findings, clinical symptoms, and differential diagnosis of DIG in patients with this central nervous system tumor.

## Introduction

Desmoplastic infantile gangliogliomas (DIG) are rare supratentorial tumors that account for 0.1-1% of childhood central nervous system (CNS) tumors [1,2]. DIG, along with desmoplastic infantile astrocytoma (DIA), is classified as a desmoplastic CNS neoplasm and has been categorized as grade I by the World Health Organization (WHO), indicating a benign tumor behavior [3,4]. Both tumors exhibit poorly differentiated cells. Histologically, in DIA, as the name suggests, the neuroepithelial cells are restricted to astrocytes. In DIG, astrocytes are associated with a variable component of neurons, known as ganglion cell components [5,6].

In terms of the clinical-radiological relationship, DIG commonly presents with macrocephaly, particularly on the side of the mass, in a frontoparietal location, causing compression and edema. This can result in bulging fontanelle in early childhood. Initial symptoms often include headache, vomiting, and seizures. Initial imaging exams, typically transfontanellar ultrasound or computed tomography (CT), reveal a solid-cystic mass, leading to sutural diastasis and occasionally bone erosion [6,7]. CT scans show enhancement of the solid component, and calcifications are frequently observed. On MRI, the cystic part appears hypointense on T1-weighted images and hyperintense on T2-weighted images, while the solid part (usually smaller) is isointense on both T1 and T2 and enhances with paramagnetic contrast along the dural surface [5-8].

DIG is considered a benign tumor with a generally favorable prognosis, and patients typically have a survival rate of 8-20 years. Complete resection of the lesion can potentially provide curative outcomes, and metastasis to the cerebrospinal fluid is rare [5,8].

## Case presentation

The patient in question is a seven-year-old boy who has been experiencing recurring headaches and vomiting in recent days. His parents did not report any previous medical issues. Initially, he was taken to the emergency room for evaluation and later referred to the gastroenterology service due to his symptoms. However, during the investigation, the patient rapidly developed amaurosis, a complete loss of vision.

**Figure 1:**
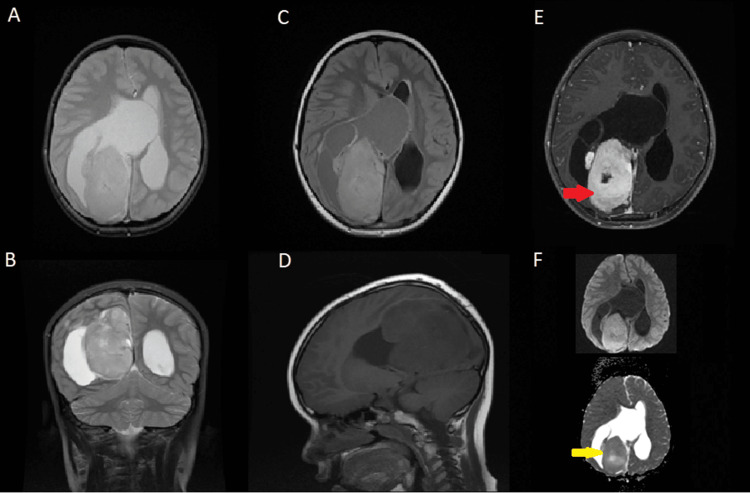
Axial (A) and coronal (B) T2-weighted images showing the solid-cystic component of the lesion with hyperintensity, centered in the parietal lobe, causing compression on the right lateral ventricle and midline deviation. Axial T2/FLAIR (C) reveals a decrease in signal intensity of the cystic component, which appears hyperintense compared to cerebrospinal fluid. Coronal T1 (D) demonstrates hypointense signal intensity on T1-weighted imaging. Axial post-contrast T1 (E) shows heterogeneous enhancement of the solid component (red arrow). DWI/ADC sequences (F) demonstrate iso-intensity of the ADC within the solid component, compared to the normal brain parenchyma (yellow arrow). FLAIR: fluid-attenuated inversion recovery; DWI: diffusion-weighted imaging; ADC: apparent diffusion coefficient

A CT scan, carried out at the originating facility, unveiled an expansive tumor within the CNS. Further characterization of the lesion was accomplished through magnetic resonance imaging (MRI) at our institution, wherein we executed pre- and post-contrast T1 sequences, T2/fluid-attenuated inversion recovery (FLAIR), and diffusion-weighted imaging (DWI)/apparent diffusion coefficient (ADC). These sequences discerned a substantial lesion situated in the right cerebral hemisphere, encompassing both cystic and solid constituents (Figure 1).

The patient was then referred to the neurosurgery service, where he underwent total resection of the tumor. Histopathological analysis confirmed the presence of a glioneuronal tumor, leading to a diagnosis of DIG.

Fortunately, the patient's recovery was uncomplicated, and upon leaving the hospital, he was scheduled for follow-up imaging. Over the course of the first two years, he remained asymptomatic, with no evidence of new lesions observed in the MRI scans.

## Discussion

DIA and DIG, previously regarded as distinct entities, are now classified together in the current (2021) WHO classification of CNS tumors. This classification acknowledges the clinical, radiological, and pathological similarities between the two conditions [9].

Typically, these tumors are in the supratentorial region and with dural-based. They are characterized by a combination of large cystic and solid components and tend to present in the early years of life, typically before the age of two years [4,10]. The most common presentation is a rapid enlargement of the head circumference, and symptoms typically become evident within a relatively short timeframe [11].

Regarding the most common imaging characteristics on MRI, we can highlight its supratentorial and intra-axial location, with solid-cystic components. The solid component typically appears isointense to the surrounding brain parenchyma on T1-weighted and T2-weighted sequences, with intense enhancement after paramagnetic contrast administration [8]. Additionally, there is the possibility of enhancement of the cystic wall and the solid portion in contact with the dura mater, potentially displaying a dural tail sign. Vasogenic edema may be present. On the ADC map, the solid tumor component usually demonstrates iso-intensity compared to the surrounding parenchyma, indicating findings consistent with a low-grade pediatric tumor [12].

Possible differential diagnoses based on imaging findings include primitive neuroectodermal tumor (supratentorial), atypical teratoid rhabdoid tumor (supratentorial), choroid plexus carcinoma, and supratentorial ependymoma [12].

## Conclusions

DIGs are rare intracranial tumors that predominantly occur in children. They exhibit distinct imaging features, such as supratentorial location, solid-cystic components, and intense enhancement with paramagnetic contrast. They also show iso signal on the ADC map compared to normal brain parenchyma, which supports a low-grade neoplasm. The differential diagnosis includes other pediatric intracranial tumors with predominantly supratentorial heterogeneous patterns. Recognition of these imaging characteristics is crucial for accurate diagnosis and appropriate treatment planning.
